# “I Feel like the Virtual Reality Really Gives You that Power Back”: A Focus Group Study Exploring Breast Cancer Surgery Patients’ Experiences with a Preoperative Virtual Reality Intervention

**DOI:** 10.1089/jmedxr.2024.0042

**Published:** 2025-01-21

**Authors:** Gabrielle S. Logan, Jada Benedictson, Kristin Reynolds, Kailey Penner, Renée El-Gabalawy

**Affiliations:** 1 Department of Anesthesiology, Perioperative and Pain Medicine, University of Manitoba, Winnipeg, Canada.; 2 Department of Clinical Health Psychology, University of Manitoba, Winnipeg, Canada.; 3 Department of Psychology, University of Manitoba, Winnipeg, Canada.; 4 Department of Psychiatry, University of Manitoba, Winnipeg, Canada.; 5 Department of Psychology, University of Calgary, Calgary, Canada.; 6 CancerCare Manitoba, Winnipeg, Canada.

**Keywords:** anxiety, breast cancer, virtual reality, perioperative, qualitative, surgery

## Abstract

Patients undergoing oncological surgery often experience elevated anxiety, partly due to the novel hospital environments (e.g., operating room [OR]). Virtual reality (VR) may be a practical and immersive way to expose patients to such triggering environments before surgery to address this anxiety. However, patient perspectives are often underrepresented in the development of these interventions, and patient feedback is essential to ensure their relevance and utility. A randomized clinical feasibility trial investigated the use of a preoperative VR OR prototype intervention with breast cancer patients undergoing surgery. Participants who indicated interest in participating in related studies were invited to take part in either an in-person (for treatment as usual [TAU] to trial the VR OR postsurgery) or a virtual (previously received preoperative VR intervention) focus group. These were audiorecorded, transcribed, and analyzed using reflexive thematic analysis. The primary objectives were to understand patients’ experiences with the VR intervention and gain patient feedback for future VR and study development. Ten participants from the feasibility study participated in one of four focus groups, six from the intervention group and four from the TAU group. Themes identified across focus groups included: (1) individuality shapes the VR experience, (2) wanting to know what to expect, (3) “a little more in charge of what’s going on,” and (4) “perspective of being on the (operating) table.” The VR intervention was generally viewed positively and seen as a valuable resource. The focus groups were useful in deciphering patient suitability through discussion of individual differences and highlighting the benefits of the intervention, such as education, exposure to the OR, and facilitating a sense of autonomy and empowerment. While participants endorsed the VR intervention’s current utility, they also provided meaningful suggestions for enhancement based on their lived experience. As new technologies such as VR are developed, it is essential to incorporate patient perspectives to ensure that these innovations effectively meet patient needs.

## Introduction

Although not yet fully integrated into health care settings, virtual reality (VR) and extended reality technologies have considerable potential for improving patient experiences for those facing a variety of health concerns.^1^ In particular, VR interventions that focus on the patient journey (i.e., fully immersing and pre-exposing participants to the perioperative experience) show promise in reducing distress and anxiety (referred to as anxiety throughout) and show high acceptability among patients.^2–4^ For these technologies to be effectively implemented into standard practice, feasibility studies that evaluate whether an intervention is realistic, practical, and useful are important and integral first steps.^5^ A feasibility study facilitates an iterative process whereby participants and other partners may provide feedback and inform changes before a large-scale randomized clinical trial (RCT) is conducted.^5^ Within feasibility research, it is recommended to gather both quantitative and qualitative evaluations on the potential impact and the acceptance of the intervention.^6^ This is particularly important when working with individuals with unique lived experiences and needs, such as cancer surgery patients. A cancer diagnosis comes with a variety of stressors; however, cancer surgery is a particularly common source of preoperative anxiety.^7^ Research has shown that the operating room (OR) environment is a particularly anxiety-inducing trigger for surgical patients.^8,9^ There is a gap in the literature examining the feasibility and use of VR as an anxiety reduction and/or prevention method that may protect against negative postoperative outcomes in individuals with cancer, particularly breast cancer.^10^

A total of 247,100 new cancer cases are expected to be diagnosed in Canada in 2024.^11^ Of these, 30,800 will be breast cancer, which is most commonly treated with surgery.^12^ Cancer surgery patients experience clinically significant rates of preoperative anxiety related to a range of factors, ranging from 23% to 77%.^13–15^ This anxiety is often associated with a range of poor perioperative outcomes including increased pain, nausea, discomfort, fatigue, and analgesic consumption.^16–20^ However, since elective surgery is a scheduled event associated with predictable physiological and psychological stress, it is an important target for preemptive stress management. While preoperative educational and counseling interventions seek to improve psychological and physical functioning through the establishment of realistic expectations of the surgical process,^21–24^ there is conflicting evidence regarding their effectiveness. In attempts to identify an alternative intervention to decrease preoperative anxiety and mitigate adverse perioperative health outcomes, researchers have examined the impact of allowing patients to tour the OR, prior to surgery.^25^ Although this intervention has been associated with reductions in levels of preoperative anxiety,^25^ it has limited feasibility to be administered broadly due to infrequent availability of ORs and limited resources and personnel to implement this intervention.

A more contemporary intervention demonstrating initial promise in reducing anxiety and other psychiatric symptoms in other contexts (e.g., fear of flying) is the use of VR.^26–29^ VR has also been gaining popularity in other medical contexts and is now being used for pain management^30^ and cognitive and physical rehabilitation.^31,32^ Using VR interventions is advantageous in health care systems because it is relatively low cost, utilizes portable equipment that can be used repeatedly in different settings, and can be translated into multiple languages and adapted across surgery types.^3,33^ Recently, researchers have evaluated whether preoperative VR simulations that expose patients to the OR can mitigate stress and anxiety,^2,34^ and these studies provide preliminary support for the efficacy of such interventions. However, this type of intervention has not yet been evaluated with patients scheduled to undergo oncological procedures. This highlights the importance of conducting feasibility studies within this population to better understand patient needs and perspectives.

Indeed, with the few studies that exist, there has been little effort to gain patient perspectives on the utility and development (or refinement) of preoperative VR interventions,^2,34^ despite the many noted benefits of incorporating people with lived experience in research.^35–37^ Importantly, research has pointed out that the involvement in intervention codevelopment can lead to health improvements in patients (e.g., recovery from illness),^38^ increased feasibility in studies (e.g., increased enrollment in interventions and improvements in trial retention),^35,38–40^ acceptability (i.e., usability and alignment with patient values and needs) metrics, relevance (i.e., applicability and importance to patients), and rigor (i.e., enhanced data quality).^35,39,41^ These outcomes highlight the importance of developing interventions using patient feedback and perspectives.

### Research objectives

The present study represents an adjunct investigation related to a randomized feasibility trial (publication of feasibility metrics is in progress) that explores the acceptability and feasibility of a novel VR OR prototype intervention for patients undergoing breast cancer surgery. Following participation in this trial, participants were postoperatively invited to participate in an additional focus group exploring patients’ experiences with the VR. Thus, the goal of this focus group study was to comprehensively understand breast cancer surgery patients’ experiences with, and the acceptability of, a preoperative VR prototype intervention. It additionally aimed to inform modifications and enhancements to the VR simulation and study design for the future RCT. Focus groups were chosen to address the qualitative aims as they allow for guided discussion, whereby participants may also build upon each other’s ideas (commonly referred to as “piggybacking”).^42^ This group discussion facilitates a more complex and nuanced understanding of the question at hand and thus may be particularly useful for understanding patient needs.

## Methods

This study was approved by the University of Manitoba Health Research Ethics Board (HS26054). Study reporting followed the Standards for Reporting Qualitative Research checklist.^43^ An initial protocol including the inclusion of these focus groups in the larger research program has also been published.^44^

### Procedures

**FIG. 1.**
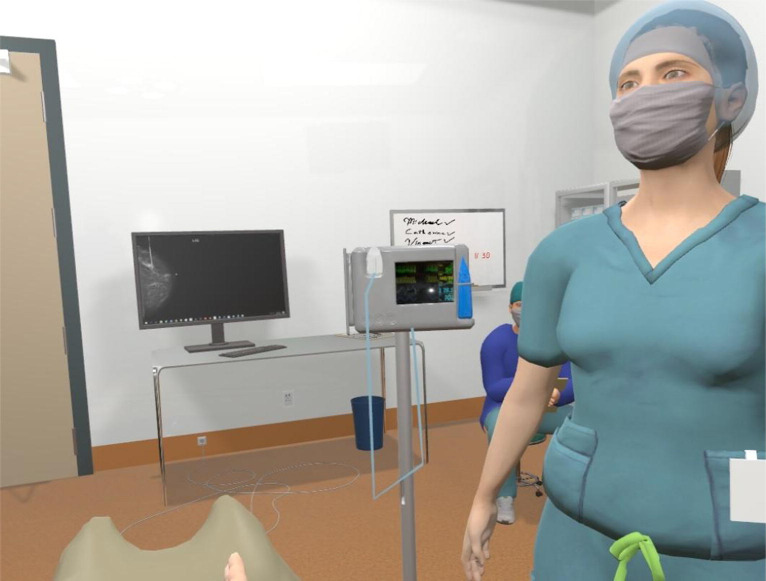
Image of VR prototype. VR, virtual reality.

**FIG. 2.**
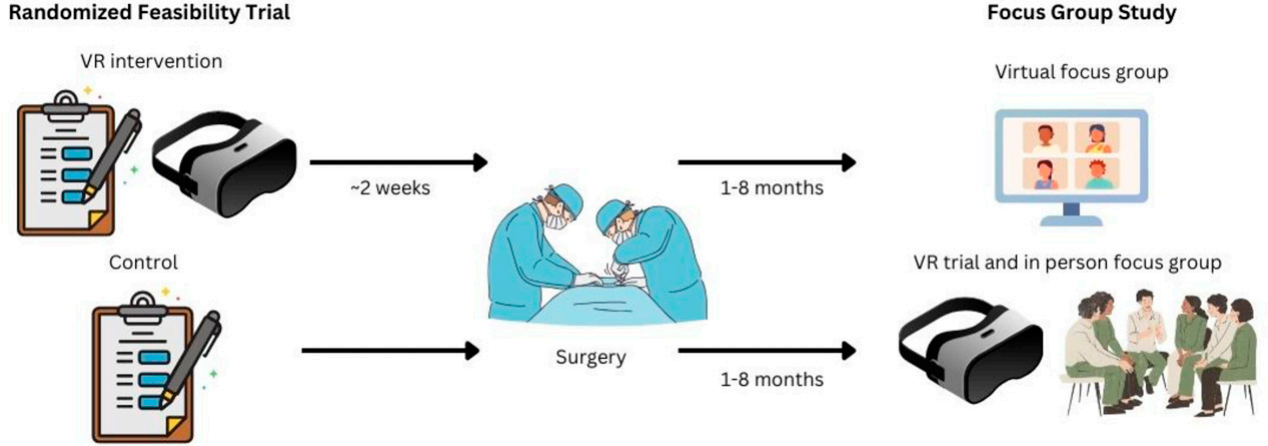
Visualization of the current study flow. *Note.* The left side represents the randomized feasibility trial (results not included here) and the right is the focus group portion, which is the focus of the present study.

A novel VR OR intervention was trialed with a series of patients undergoing breast cancer surgery (publication in progress; see ref. [44] for protocol). Participants in the primary feasibility trial were randomized to either treatment as usual (TAU) or a single session exposure of the VR OR intervention 1–2 weeks prior to surgery. Briefly, the VR OR intervention allowed participants to explore a simulated OR and go through an induction experience firsthand (see Fig. 1 for an image of the prototype). For more information about the development of the VR, please see the protocol.^44^ One to eight months postrandomization, we conducted focus groups as a follow-up (see Fig. 2 for a visualization of the current study flow).

Of all participants who participated in the feasibility trial, regardless of randomization, those who indicated that they would be willing to participate in a patient advisory group and future research projects were contacted by phone by research staff and invited to participate in focus groups. Participants had undergone cancer surgery at a large academic medical hospital in central Canada (Health Sciences Centre in Winnipeg, Manitoba), were over the age of 18, and could speak/read English. Interested participants were emailed a consent form to complete before participation. Participants included in the focus groups were from both the intervention group (Focus Group A in the current study) and the TAU group (Focus Group B in the current study). All participants received an honorarium for their time.

Focus group facilitation was conducted by the study authors (G.S.L., J.B., and K.R.). Focus group questions were created by the team alongside one of the authors (K.R.), who has extensive qualitative experience (see Supplementary Data for focus group questions).

### Focus Group A

Individuals from the VR intervention group in the original trial were asked to take part in a virtual focus group over Zoom (Version 6.0.11). To accommodate all participants in this group, two virtual focus groups were held, with at least two facilitators present at each group. One facilitator asked semistructured question prompts, and the second facilitator ensured that the technology ran properly, took field notes, and asked probing questions as needed. Groups were audiorecorded and transcribed using within-software functions and then checked for accuracy and deidentified. Virtual focus groups lasted an hour.

### Focus Group B

Individuals in the TAU group in the original trial were invited to one of two in-person focus groups. Immediately prior to the focus groups, all TAU individuals were asked to trial the VR OR intervention, which took approximately 5–10 min per person. Subsequently, the focus groups began following TAU-specific semistructured prompts. These focus groups were audiorecorded using a voice recorder and transcribed using Trint software, deidentified, and then checked for accuracy. Trint is a transcription software approved by our university ethics board that utilizes strict information security standards. Furthermore, across both focus groups, no names were collected to ensure participants could not be identified via audio. In-person focus groups lasted between 1 and 1.5 hours.

Focus groups needed to be in-person for the TAU group (Focus Group B) so that they could trial the intervention prior to participating in the focus group. In contrast, focus groups were held virtually for the intervention group (Focus Group A) to allow for greater flexibility and ensure continued participation, given that in-person requirements can be a barrier to participation. See Figure 2 for a visual depiction of study flow.

### Data analysis

Focus group qualitative data were analyzed using reflexive thematic analysis (RTA).^45–47^ RTA was chosen because it is theoretically flexible and may attend to questions about one’s experiences, views, and perceptions.^45^ There is also flexibility in how the data are approached,^45^ and researcher subjectivity is seen as a benefit rather than a source of bias.^46^ RTA requires prolonged engagement with the data, so that themes may be generated and reflected upon cyclically.^46^ Research personnel G.S.L. and J.B. were both involved in the coding process. Since RTA is interpretative, it generally does not require multiple coders; however, here, this collaboration was based on discussing interpretations to develop deeper meaning rather than comparing codes to reach a consensus.^48^

We adhered to all six phases of RTA outlined by Braun and Clarke, which included (1) data familiarization, (2) data coding, (3) initial theme generation, (4) reviewing and clarifying themes, (5) finalizing theme names, and (6) writing the report.^47,49^ The researchers began by familiarizing themselves with the data, carefully reading through each focus group transcript, and taking general notes or making observations. The audio files were also referenced at this time to supplement reading over the transcripts. Additionally, any field notes that were taken during each of the focus groups were also reviewed for further context. Once well-acquainted with the focus group data, the researchers independently coded the first transcript by highlighting and adding comments. As described in Braun and Clarke,^45–47^ this step involves documenting brief codes to the transcript that succinctly capture all core ideas and concepts that emerge from the data. This involves providing labels (i.e., comments) that make sense of the data in relation to the research questions and that can be understood without looking back through the transcript. Eventually, these codes will either come together to produce themes or be discarded if not relevant to the research question.^48^ Coders completed this process in Microsoft Word for ease of navigating through codes as well as comparing independent coding documents for discussion. This process was repeated for all four transcripts, with the researchers meeting between each to discuss codes leading to theme generation. However, as described by Braun and Clarke, the process was nonlinear and iterative, with themes and codes continually being reworked as the researchers’ understanding of the data evolved.^47,49^ G.S.L. and J.B. also met with the larger study team to review drafts of the qualitative framework and obtain feedback from the lenses of all researchers before finalizing the framework for the final stage of producing the written report. In line with the constructivist and reflexive nature of RTA, the richness and quality of the data were used as a marker of data completion, as opposed to other metrics such as data sufficiency or saturation.^50^ In each main theme and subtheme, we have documented strong and supportive quotes, highlighting the alignment of the data with the resulting thematic framework.

### Positionality statement

When conducting reflexive qualitative research, it is important to consider the identities of the researchers that approached the data. Both G.S.L. and J.B. are Canadian women with a postsecondary education. They have been heavily involved in this study, including participant recruitment, data collection, and analysis for the initial feasibility study. As a result, they have been involved with these participants both prior to and following their cancer surgery, a sensitive and major life event. However, both researchers recognize their understanding of these patients’ experiences is limited, having not personally experienced a major diagnosis or surgery of this kind. Throughout this process, G.S.L. and J.B. gained considerable experience and proficiency with VR technology, whereas this was many participants’ first exposure to VR. Evidently, it is important to consider the researcher’s positions of privilege and how their health and educational experiences may have influenced the interpretation of the data. Despite this, there were efforts taken to maintain closeness to the data. First, using two coders enhanced accountability and ensured that interpretations remained aligned with the data. The researchers also grounded themselves in other breast cancer patients’ experiences from previous work within the lab.^51^

## Results

Of the 23 participants who participated in the feasibility study, 10 were successfully contacted and consented to participate in one of the focus groups. Six participants had been previously randomized to the VR OR intervention group in the feasibility study. One of these participants did not view the VR before surgery as intended due to a technical issue with the VR (i.e., the VR system required the computer to be updated). As a result, the research staff opted to simply show the participant some visuals of what the VR OR looked like from the computer. Since this participant did not get to fully experience the VR intervention, they were invited back to view it before the scheduled virtual focus group. Four participants were previously randomized to the TAU group. Participant demographic information was collected with other baseline measures as a part of the feasibility study either at their VR intervention visit or on their day of surgery (TAU). Participants were female and were aged between 34 and 66 (*mean* = 47.9, *standard deviation* = 9.7). All participants had received at least some college education or higher, and 80% were either married or in a common-law relationship. Eight (80%) of the participants had previously received surgery before for a different condition (e.g., tonsillectomies and c-sections), but none had received breast cancer surgery. Most participants were scheduled to receive a single mastectomy with reconstruction (40%), a double mastectomy with reconstruction (30%), or a lumpectomy (30%). Further details on sample characteristics are summarized in Table 1.

**Table 1.** Focus Group Sample Characteristics

Demographic	*n*, % (*N* = 10)
Age, *M* (*SD*)	47.9 (9.7)
Female, *n* (%)	10 (100%)
Education	
Some college	1 (10%)
College degree or higher	9 (90%)
Marital status	
Married/common-law	8 (80%)
Divorced	2 (20%)
Previous surgery	8 (80%)
Breast cancer surgery type	
Lumpectomy	3 (30%)
Single mastectomy with reconstruction	4 (40%)
Double mastectomy with reconstruction	3 (30%)

M, mean; SD, standard deviation.

### Themes

#### Theme 1: Individuality shapes the VR experience

A central theme across the focus groups was participants’ perceptions of the suitability and impact of the VR OR for both themselves and others. Although all participants shared having a breast cancer diagnosis requiring surgery, there was an understanding that everyone had different life and cancer circumstances and unique needs. This led to a consideration of factors that may affect engagement with the VR intervention. Participants primarily grounded the perceived utility of viewing the VR OR through the lens of their own past surgical experiences and personalities/emotional states.

#### Subtheme 1: First surgical experience

The novelty of the OR was reported as a key feature that may impact one’s interest in the intervention. One’s level of familiarity with the OR may impact engagement at multiple levels, from agreeing to participate in the intervention to the attention given to actively exploring the VR OR environment. Thus, not only may past surgical experiences influence suitability but also the degree of impact the VR OR may have on the viewer. For example, participant 2 explained, “*I found that the VR was more for maybe somebody who has never had surgery before to go through it.*” Participant 3 corroborated this by saying “That *was sort of my reason or interest, for it [VR]. Was that it was my absolute first time having any surgery done whatsoever*.”

In a discussion of suitability and impact, it is important to note that two participants from the TAU group who viewed the VR OR intervention postoperatively reported negative experiences. These participants (i.e., 5 and 8) described the experience as “really triggering” and “scary.” Despite this, they also both endorsed feeling as though viewing it preoperatively would have been beneficial to them.

#### Subtheme 2: Personality traits and emotional states heading into surgery

Participants reported approaching their cancer journey and surgery with varying emotional states, as influenced by their unique personality traits. This ranged from individuals with “*very high anxiety*” (participant 5) to those who felt “*pretty calm and in control of [their] emotions*” (participant 4), which was endorsed as something that may affect VR OR suitability. For example, participant 6 stated, “*I’m not an anxious person, so my thought automatically goes to the people that do err or in the anxiety side right now, because me or someone who’s not at all anxious, I tend to be like a very like…serenity now and in my own zen… personality… Like my daughter is extremely anxious. I think that their levels would have been so high on surgery day*.”

#### Theme 2: Wanting to know what to expect (preparation)

A primary benefit noted by many of the participants was that the VR OR provided an idea of what to expect on the surgery day. A cancer diagnosis comes with many “unknowns,” which participants described as the source of their “*tension*” and “*anxieties*” because they were often “*not sure what was going to happen*” (participant 2). Although the VR OR could not clarify all their uncertainties, it appeared to fill one significant gap in their knowledge—what to expect in the OR environment. Specifically, it allowed participants to gather information about the OR and the surgical procedure while also exposing and thus familiarizing them with the OR environment.

#### Subtheme 1: Information gathering

Participants largely endorsed the idea that the more information you receive, the more prepared and informed you will feel. This sentiment was echoed by participant 5 stating “*Who wouldn’t want more information or education*?” Participant 6 took it further speaking to how “*…the more information you can give patients, the better they’re going to feel, the less they’re going to have anxiety.*” The ability to gather information on OR elements and procedures enhanced preparedness and was even described as “*beneficial*” (participant 9), “*helpful*” (participant 7), and “*fun*” (participant 4).

#### Subtheme 2: Addressing the unknown and familiarization

Exposure to the VR OR environment prior to surgery was noted as beneficial within itself. Participants shared that it gave an idea of “*what I can expect*” (participant 5), took “*some of that guesswork out*” (participant 7), and helped in “*bringing that reaction down*” (participant 2) on the day of surgery. Participant 4 summarized this by saying “*Once I got into the OR, then it did remind me very much of the VR. And it did put me at ease when I’m laying there and waiting for them to do their thing. It was like, oh, okay, this is familiar.*” Additionally, being immersed in the VR OR also gave participants “*permission to look*” (participant 9) where they may not have otherwise been able to look around the OR on the day of surgery.

#### Theme 3: “More in charge of what’s going on.”

A third theme identified was how the VR OR gave participants a sense of “*control*” or feelings of autonomy over their cancer journey. Many patients described feeling that factors associated with cancer were often out of their “*control*” but that the “*VR really gives you that power back*” (participant 5). This stemmed from both being able to take things at their own pace (within the VR OR) and through being given the opportunity to make choices surrounding their care.

#### Subtheme 1: Taking it at your own pace

A primary benefit noted by participants was that it gave them the opportunity to explore the OR room at their own pace, as the VR OR experience began with open time for participants before the structured induction component. Participant 5 spoke to the utility of this by saying:

“*I took my time at my own pace to look I’m like k, and then I swung back and like oh you’re not ready. Then I went back. But if we’re in an operating room I can’t ‘guys give me 10 minutes?’ I want to like enjoy the room like, no, we got to do surgery, we’re timed. So I really enjoyed, like, the pace that I could do it on my own terms*.”

Additionally, once the structured part of the intervention began, participants could further explore the room and the tasks being performed by the health care professionals. Participant 1 explained:

“*Here you could just slow it out, down, look around as long as wanted, and study that pretend real machine, look at other doctors. And what are they doing? Isn’t that interesting, right? So you could, you could take it at your own pace. So I guess that’s the benefit, right?*”

#### Subtheme 2: Making decisions about care

Many participants endorsed the idea of being able to make choices based on their individual needs. Regarding choosing to participate in the intervention at all, participant 1 noted “*But I mean if you have it as a choice for people. If it’s an option. Those who want it [VR] or need it will take advantage of it*.” Furthermore, the VR OR participants are given a choice over how they spend their time and what they look at. For example, “*when you were in the room and you had those minutes to look around, you could focus on a certain place… like, if I really was focused on the heart rate monitor or something right? Like, I really wanted to know the ins and outs of it*” (participant 10). This ability to have choices or options inherently appeared to have provided autonomy. Participant 10 explained, “*We feel like a little more in charge of what’s going on instead of you being the person on this bed*.”

#### Theme 4: “Perspective of being on the [operating] table.”

A fourth and final theme came from the benefits of the VR technology within itself, the unique and meaningful perspective and experience it was able to provide. The VR technology was commented on particularly in the context of providing a first-person perspective and the adaptability of realism and graphics within VR.

#### Subtheme 1: First-person perspective

Participants highlighted the first-person perspective as “*very helpful*” and “*important*” (participant 3). As participant 6 noted, “*because it’s first person, it just brings it to a different level*.” Participant 3 summarized this experience by saying:

“*I think it was the perspective of being on the table and looking around and having the others like having the doctors and nurses focusing on you. And just slightly different than like watching a show and seeing or walking past a room. So it was the experience of actually being the one in the chair I think that helped, just the perspective of it*.”

#### Subtheme 2: Adaptive graphics and realism in VR

Participants frequently commented on the graphics and degree of realism within the VR OR. Although the overarching consensus was that the VR “*did give a picture of what it was really like*” (participant 4), there were some differences in opinion. Participants 8 and 5 reported “*It [VR] does feel real*” and “*I was like, ‘wow’ I’m back in the OR*,” whereas other participants, such as participants 2 and 3, described the VR OR as “*like a game*” or “*dreamlike*.” Both perceptions may be useful and demonstrate that the VR OR accurately depicted the real OR but also still provided a degree of separation.

The attention given to noises and background features within the VR also appeared to add to the realism. Participant 5 recalled how the “*beep beep beep*” and “*the hustle and bustle and lots of metal noises*” made it “*pretty spot on*” to the actual OR. Participant 6 overviewed the importance of this by saying, “*But I think the VR really just allows you to experience it in a much more real realistic way. It’s one thing to tell someone what’s going to happen; Yeah, it’s another thing to see it*.”

Another noted benefit of the adaptability of this technology was how the VR OR could provide a more calm and orderly representation of the real OR. Participant 8 described the VR as: “*it’s more calmer. You know, it’s not that busy in here… Like, compared to the real one*.” The customizability of the VR technology allowed the OR to approximate the real OR without the element of “chaos” apparent in the real OR. The lower intensity of the room was received positively, with participant 5 exclaiming “*it was really nice that it was like methodical and literally I felt like I was back in room in the operating room step by step*.” Evidently, the immersive and adaptive qualities of VR technology allowed participants to experience an environment that was both visually and auditorily realistic while also controlled.

### Recommendations for VR and future RCT

Another important element of conducting these focus groups was to gather feedback and insights in order to further develop the next iteration of the VR OR program and inform future RCTs. Participants’ lived experiences allowed them to identify what was helpful but also what could be added or changed to enhance the utility of the VR intervention and to design a more effective study in the future.

Participants provided numerous ideas on how the VR intervention may be adapted to make it more like the real experience. However, there were a few key ideas that were more strongly and widely endorsed. Many participants noted how there were fewer people present in the VR OR as compared with the actual OR, with participant 6 stating, “*The first thing I said was like there were way more people in my surgery than what was in the VR.*” Accordingly, for some participants, it seemed that including more people, “*just more hustle and bustle, more noise, more chaos*” (participant 6), would be pertinent to the VR OR experience, making it more like the real OR. Despite the VR OR also having an overhead light, participants commented on how this should be more emphasized, as it was “*massive*” (participant 5) and “*brighter*” (participant 7) in reality. In line with the theme of preparation and valuing as much information as possible, many participants also endorsed wanting to see more of the surgery day environment, with participant 7 noting, “*once you’re in there, like all that information in the VR was really helpful, but it’s only about half of the experience.*” Potential additions noted by participants included the preoperative holding area, the hallway leading up to the OR as “*that’s kind of the big reveal*” (participant 3), and even waking up in the postoperative room.

In addition to participants explaining their ideas, feedback was gathered on specific ideas that had emerged from the main feasibility study (see Supplemental Material for the specific elements that were inquired about), notably on the inclusion of a relaxation strategy led by a health care avatar. In response, participant 6 explained:

“*I think that would be helpful to some people. I wouldn’t. I don’t know if I would suggest making it like part of the VR, but something that people could click on or opt into. If the, you know this some people I mean, like, I don’t I don’t want to learn to deep breathing I just want to get this over with where some of you might find them very therapeutic for your mental health*.”

This same sentiment was echoed in response to making elements of the VR OR interactive (such that participants could click on machines or avatars with the controller and learn what they are/what they do). Although both of these elements were endorsed as useful, the ability to opt in or out of different parts of the intervention seemed preeminent.

Lastly, there was also discussion about the study location and whether the VR OR would be useful at home (for clarity, the intervention was only trialed in person at the Health Sciences Centre for the current study). Many participants felt coming into the hospital for the intervention was useful as “*if you’re going to be doing a virtual reality to take you through the experience, it makes sense to sort of be in the environment where that’s going to happen, because that’s part of the anxiety, too*” (participant 7). Indeed, participants stated that it would also prevent participants from having to deal with any technological challenges on their own. However, participants did think that “*something as simple as even just having… videos of the operating room in conjunction with the VR, … could be very helpful in terms of people’s anxiety and stress levels before surgery*” (participant 6).

Participants were also encouraged to provide recommendations for future RCTs. Participants were primarily consulted on the timeline, level of commitment, and length of the VR intervention. Patients were overall satisfied with their experience in the research study itself, noting the timeline was appropriate and the length of the intervention was suitable. Given that many participants also had numerous weekly appointments which the 30-min VR intervention could be stacked onto, the intervention was not seen as a burden. Some participants suggested reconsidering or adapting the measurement scales used in the study. For example, instead of focusing on anxiety and distress, they noted it may be beneficial to assess positive outcomes, such as how comfortable or prepared participants felt. Participants felt that this shift in focus could provide more relevant data on the VR program’s effectiveness in improving patient experiences.

## Discussion

The purpose of the present study was to gather breast cancer patients’ experiences using a novel preoperative VR OR intervention designed to reduce surgery anxiety. Across four focus groups, four main themes were extracted. These themes touched on intervention suitability and impact, how VR provides a sense of autonomy and helps enhance feelings of preparedness, as well as important features of the technology within itself being first-person and engaging. Based on their lived experiences, participants were also welcomed to provide insight for future iterations of the VR OR intervention and an eventual RCT. This study is the first of its kind to evaluate patient experience with a VR OR intervention not only to understand its current acceptability and feasibility but also to advise a subsequent patient-informed version.

Overall, participants generally agreed that the intervention would be particularly useful for those undergoing their first surgical experiences, as those who had already undergone surgery under a general anesthetic may not have found the intervention to be quite as impactful. This aligns with previous research that has shown that a history of surgery has been related to feeling safer^52^ and feeling less anxiety^53^ prior to surgery. Indeed, patients with cancer often experience uncertainty and fear of the unknown regarding their treatment progression and other factors;^51^ thus, it is not surprising that patients who have already undergone surgery often feel less anxiety with having been already exposed to the OR room and surgical experience.

Additionally, several participants agreed that the intervention would be suitable for those who have elevated anxiety, as it would help prepare them as much as possible for their surgical experience. Indeed, this is supported by research that shows that touring the OR prior to surgery in person^25^ or via VR^34^ can have significant impacts on anxiety reduction presurgery. The underlying theory is that patient anxiety is reduced by making the unknown (e.g., the OR and the experience) familiar.^54^ As such, prehabilitative VR interventions for people with cancer show promise for those undergoing their first surgical experience and for highly anxious individuals. However, future research is still needed to determine their effectiveness.

Furthermore, many participants described that cancer is a disease that often takes away personal autonomy and control, which is consistent with previous research.^51^ As such, several participants described the VR intervention as being a source of empowerment for being able to take some control back and feel autonomous. Participants were given freedom in how to spend their time in the VR intervention. A sense of empowerment for cancer patients is key, as empowerment in patients with chronic illnesses has been associated with increased satisfaction, better adherence to treatment regimens,^40,55^ and better health-related quality of life.^56^

Several participants highlighted that VR provides a unique opportunity where the patients themselves are at the core of the experience, providing a first-person perspective that distinguishes it from other interventions and potentially enhancing outcomes. Many preoperative interventions consist of simple information dissemination (e.g., pamphlets and websites) or the use of some sort of media to demonstrate to participants what will happen (e.g., showing videos of someone else undergoing similar procedures).^57^ However, the current intervention allows participants to feel as if they are in the situation, creating a sense of familiarity once they reach the actual operation, which, in turn, is associated with reduced anxiety.^54^ Moreover, the immersive quality of VR is also physiologically supported, with studies showing that immersing patients in these stress-inducing situations may alter biomarkers of stress.^58^

### Limitations and future research

This study should be considered in the context of a few limitations. First, the intervention aims to only target one form of state or situational anxiety, and it is well recognized that preoperative anxiety can relate to a number of different factors (e.g., body image and recovery).^51^ However, it is important to focus on feasible intervention targets to reduce different forms of state anxiety. These results will also inform the development of the current prototype to add additional elements that will serve to further reduce other forms of state anxiety, as highlighted in the recommendations for the VR section. Second, ethnicity was not collected, and we therefore do not know if we had a biased or generally diverse sample. As a result, these findings are not definitive and would not reflect all understandings of the experiences shared within this study. Moreover, some individuals who had experienced advancements in their cancer postsurgery or who were still undergoing treatment declined to participate. If only those who experienced more positive treatment outcomes chose to participate, this could have skewed our focus group sample. Similarly, those who were highly anxious or in distress may not have had the capacity to participate, potentially biasing the results. Furthermore, although largely in line with the general population,^59^ a significant portion of the sample (80%) had already undergone a previous surgery, and based on focus group feedback, those without surgical histories may have been the most suitable for this experience. Consequently, we may have missed more diverse perspectives of those undergoing surgery for the first time. That being said, given that the majority of individuals experience more than one surgery in their lifetime,^60^ it is important to hear perspectives that the current intervention does have on those who are undergoing another type of surgery. Furthermore, the sample was highly educated, which is not uncommon in clinical trial research since individuals with lower education levels often face greater barriers to research participation.^61^ Future focus groups should place specific emphasis on recruiting diverse and minoritized groups following recommendations by Hamel and colleagues on Multilevel Intervention Models.^62^ This is essential in ensuring that the voices of diverse and minority populations are heard, and their needs are integrated into interventions that apply to them. Taken together, the sample is not representative of the full breast cancer surgery population in Canada and should inform future recruitment targets for clinical trials and obtaining diverse perspectives.

Additionally, although research highlights that content that emerges from both in-person and virtual focus groups is highly similar, there may have been some nuanced differences in the interview format that would have given us different results.^63,64^ Importantly, due to the variation in follow-up points (e.g., 1–8 months), there may have been important information missed or skewed due to recall bias. Finally, although this sample size is consistent with other studies using RTA,^65–67^ a larger sample size may have led to more themes. However, consistent with RTA theories, data quality is more important than data quantity.^50^

Despite these limitations, this study gathered important information on patients’ perspectives and recommendations for VR intervention development. Patient voices are often neglected or seldom incorporated,^35–37^ despite the noted benefits to both participants^38,40^ and the research itself.^35,38–40,68–70^

## Conclusions

In this qualitative focus group study gathering feedback on a preoperative VR OR prototype intervention for patients undergoing breast cancer surgery, participants highlighted the potential positive impact for those without a history of surgery and those who are highly anxious. Many participants highlighted how the intervention provided a sense of autonomy and put them at the center of the experience. Furthermore, a series of patient-driven recommendations for future VR prehabilitation interventions were provided, which will be incorporated into the development of the next version of the prototype and inform other research. VR interventions have very important advantages. These interventions are adaptable, have the flexibility to be carried out across various settings (e.g., home, hospitals), require minimal to no personnel, and can be patient-operated.^71^ They are additionally simpler compared with conventional interventions and are resource and time-efficient,^3,33^ all of which make VR interventions highly scalable and an important avenue to keep exploring. RCTs incorporating patient-oriented feedback are needed to determine the efficacy of this particular intervention within cancer populations.
